# Chemosensetizing and cardioprotective effects of resveratrol in doxorubicin- treated animals

**DOI:** 10.1186/1475-2867-13-52

**Published:** 2013-05-28

**Authors:** Abdel-Moneim M Osman, Sameer E Al-Harthi, Ohoud M AlArabi, Mohamed F Elshal, Wafaa S Ramadan, Mohamed N Alaama, Huda M Al-Kreathy, Zoheir A Damanhouri, Osman H Osman

**Affiliations:** 1 Pharmacology Department, Faculty of Medicine, King Abdulaziz University, Jeddah, Saudi Arabia; 2 National Cancer Institute, Cairo University, Cairo, Egypt; 3 Princess Al-Jawhara Center of Excellence in Research of Hereditary Disorders, Jeddah, Saudi Arabia; 4 Department of Biochemistry, Faculty of Science, King Abdulaziz University, Jeddah, Saudi Arabia; 5 Molecular biology Department, Genetic engineering and Biotechniology Department, Minoufia University, Minoufia, Egypt; 6 Department of Anatomy, Faculty of Medicine, King Abdulaziz University, Jeddah, Saudi Arabia; 7 Department of Medicine, Cardiology unit, Faculty of Medicine, King Abdulaziz University, Jeddah, Saudi Arabia

**Keywords:** Doxorubicin, Resveratrol, Potentiation, Cardioprotection, Cell cycle disturbance

## Abstract

**Background:**

Doxorubicin (DOX), an anthracycline antibiotic is one of the most effective anticancer drug used in the treatment of variety of cancers .Its use is limited by its cardiotoxicity. The present study was designed to assess the role of a natural product resveratrol (RSVL) on sensitization of mammary carcinoma (Ehrlich ascites carcinoma) to the action of DOX and at the same time its protective effect against DOX-induced cardiotoxicity in rats.

**Methods:**

Ehrlich ascites carcinoma bearing mice were used in this study. Percent survival of tumor bearing mice was used for determination of the Cytotoxic activity of DOX in presence and absence of RSVL. Uptake and cell cycle effect of DOX in tumor cells in the presence of RSVL was also determined. Histopatholgical examination of heart tissues after DOX and/or RSVL therapy was also investigated.

**Results:**

DOX at a dose level of 15 mg/kg increased the mean survival time of tumor bearing mice to 21 days compared with 15 days for non tumor-bearing control mice. Administration of RSVL at a dose level of 10 mg/kg simultaneously with DOX increased the mean survival time to 30 days with 70% survival of the tumor-bearing animals. RSVL increased the intracellular level of DOX and there was a strong correlation between the high cellular level of DOX and its cytotoxic activity. Moreover, RSVL treatment showed 4.8 fold inhibition in proliferation index of cells treated with DOX. Histopathological analysis of rat heart tissue after a single dose of DOX (20 mg/kg) showed myocytolysis with congestion of blood vessels, cytoplasmic vacuolization and fragmentation. Concomitant treatment with RSVL, fragmentation of the muscle fiber revealed normal muscle fiber.

**Conclusion:**

This study suggests that RSVL could increase the cytotoxic activity of DOX and at the same time protect against its cardiotoxicity.

## Introduction

Doxorubicin (DOX) was introduced in cancer therapy in the late 1960s. It has emerged as one of the most potent broad-spectrum antitumor anthracycline antibiotics. DOX can be administered as a single agent or in combination with other chemotherapeutic agents. It is widely used in the treatment of variety of cancers, including leukemias, lymphomas, soft-tissue sarcomas, and solid tumors. Its cytotoxic effects on malignant cells, however, are complicated by an increase in the risk of cardiotoxicity [1,2].

Due to the increasing worldwide prevalence and health burden of doxorubicin induced cardiotoxicity, it has become increasingly important to find pharmacological remedies to protect against this serious side effect, in an attempt to minimize DOX effective chemotherapeutic dose and thereby its side effects, a variety of approaches have been investigated [3-5]. One of them is the search for natural compounds with chemopreventive or anticancer properties that can be used in combination with doxorubicin. Resveratrol (RSVL) (trans – 3, 5, 4 – trihydroxystilbene) is a naturaly occurring poly-phenolic compound found primarily in root extracts of the oriental plant Polygonum cuspidatum and many other plant species [6]. It is highly abundant in skins of red grapes and moderately abundant in peanuts and blueberries [6]. It has recently been discovered that it has many beneficial effects in different biological systems, which include anti-inflammatory, antioxidant, anti-neoplastic, anti-carcinogenic, anti-tumorigenic, cardioprotective, neuroprotective, anti-aging and antiviral effects [6]. Its potential chemopreventive and chemotherapeutic activities have been demonstrated in all three stages of carcinogenesis (initiation, promotion, and progression) [7]. A study from our lab used a model of DOX-induced heart damage in rats, we found that pre-treatment with aged garlic extract, a strong antioxidant, offered protection against DOX-induced myocardial cell damage [8]. Recently, Osman et al. [9] reported sensitization of human breast cancer cells to the action of DOX in an attempt to minimize DOX effective dose and thereby its side effects. Therefore The present study was undertaken to test whether resveratrol in vivo could potentiate the antitumor properties of doxorubicin and the mechanisms by which this could happen. Also could resveratrol protect against DOX-induced cardiotoxicity.

## Materials and methods

### Reagents

Doxorubicin hydrochloride and RSVL were purchased from Sigma–Aldrich Co. (St. Louis, MO, USA). The stock solution of both drugs were dissolved in normal saline and preserved at -20°C. The solutions were diluted in normal saline immediately before each experiment to the desired final concentration.

### Animals and tumor

Female Swiss albino mice (8 weeks of age, 20–25 g body weight) were obtained from King Fahd Medical Research Center, King Abdulaziz University, Jeddah, Saudi Arabia. The animals were acclimatized for one week. A commercial balanced diet and water, ad libitum were provided throughout the experiment. A line of Ehrlich ascites carcinoma cells (EAC) cells was supplied by Prof. Abdel-Moneim and maintained in our laboratory by weekly I.P. transplantation of 2.5 × 10^6^ cells/mouse. This study was approved by the ethical committee of King Abdulaziz Hospital.

### Evaluation of antitumor activity

The effect of RSVL on the antitumor activity of DOX was evaluated using the modified regimen of Donenko et al. [10]. In brief, EAC cells were inoculated I.P. into forty female Swiss albino mice (2.5 × 10^6^ cells/mouse). Twenty four hours later, mice were equally divided into four groups. Group 1: mice were administered normal saline i.p. (0.2 ml/20 gm) and served as control. Group II was administered DOX 15 mg/kg i.p. Group III received a single dose of RSVL (10 mg/kg,i.p.) and Group IV received RSVL simultaneously with DOX.

### Assessment of doxorubicin cellular accumulation

DOX cellular accumulation assessment in Ehrlich ascites cells was performed using spectrofluorometer (F-2000 Fluorescence spectrophotometer Hitachi, Japan) according to the method of Kitagawa et al. [11]*.* Ehrich ascites cells were inoculated as described above at 10×10^6^ cells/mouse. Twenty-four hours later RSVL (15 mg/kg) was injected i.p. simultaneously with DOX (20 mg/kg). Tumor samples were obtained at fixed times after DOX treatment, washed, counted, and DOX fluorescence intensity was measured at excitation and emission wavelengths of λ ex = 496 nm and λ em = 592 nm, respectively to determine DOX concentration.

$$\begin{array}{l} \mathrm{DOX}\;\mathrm{cellular}\;\mathrm{accumulation}\;\mathrm{ratio}\;\\ =\;\frac{\mathrm{DOX}\;\mathrm{concentration}\;\mathrm{in}\;\mathrm{RSVL}\;\mathrm{treaated}\;\mathrm{cells}}{\mathrm{DOX}\;\mathrm{concentration}\;\mathrm{in}\;\mathrm{cells}\;\mathrm{treated}\;\mathrm{with}\;\mathrm{DOX}\;\mathrm{alone}} \end{array}$$

### Cell cycle analysis

Ehrlich ascites cells were inoculated i.p. (10×10^6^ cells/mouse). Twenty-four hours later, DOX (20 mg/kg,i.p.) was injected simultaneously with RSVL (10 mg/kg,i.p.). T umor cells were obtained at fixed times after DOX treatment. Cell cycle analysis was performed using flow cytometer (Becton Dicknson, BD, FACSCalbur, USA) according to the method of Smets et al. [12].

### Cardio toxic effect of DOX in presence of RSVL

Twenty-five male Wistar rats were divied into five equal groups consisting of 5 animals each and housed in a room with regular 12 hr. light/dark cycle with free access to food and water. Two groups (I and II) were used as a control and received normal saline (i.p.) and RSVL (15 mg/kg,i.p.). Group III received a single dose of DOX at dose of 20 mg/kg. Groups IV received DOX simultaneously with RSVL. Twenty four hours after DOX treatment, animals were anesthetized and heart specimens were fixed in 10% formalin for light microscopic study of the heart tissues.

### Statistical analysis

Statistical analysis was performed using SPSS (statistical package of social sciences, version 16). One way analysis of variance (ANOVA) followed by least significant difference (LSD) for post hoc analysis, was used for multiple comparisons. Statistical significance was acceptable to a level of p < 0.05.

## Results

### Survival of tumor bearing mice

**Figure 1:**
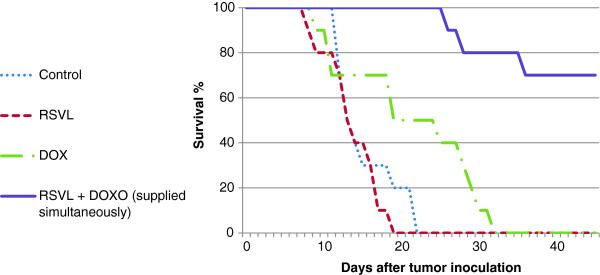
Effect of RSVL treatment (10 mg/kg,i.p.) on the cytotoxic activity of doxorubicin (10 mg/kg,i.p.) against the growth of Ehrlich ascites cells.

Table 1 and Figure 1 show the effect of treatment with DOX and/or RSVl on the survival of tumor bearing mice. Tumor-bearing control mice showed mean survival time of 15 days, whereas administration of a single dose of DOX (15 mg/kg,i.p.) increased the mean survival time to 21 days, with 10% long term survivors. Concomitant treatment with RSVL (10 mg/kg,i.p.) increased the mean survival time of tumor-bearing mice to 30 days with 70% long term survivors.

**Table 1:** Effects of RSVL treatment (10 mg/kg,i.p.) on the therapeutic action of DOX (15 mg/kg,i.p.) in mice bearing Ehrlich ascites carcinoma cells

**Treatment**	**Mean survival time**	**45-day survivors**
**Control**	15 ± 4.06 ^**a, b**^	0/10
**RSVL (10 mg/kg)**	13 ± 3.55 ^**b**^	0/10
**DOX (15 mg/kg)**	21 ± 9.19 ^**a, b**^	1/10
**DOX (15 mg/kg) + RSVL (10 mg/kg) (supplied simultaneously)**	30 ± 5.29 ^**a, b**^	7/10

Each data represents the mean ± S.D. of ten mice. The mean Survival time is the time in days until animals die.

^**a**^Significantly different from control at P-value ≤ 0.05. ^**b**^Significantly different from DOX at P-value ≤ 0.05.

### DOX level in tumor cells

**Figure 2:**
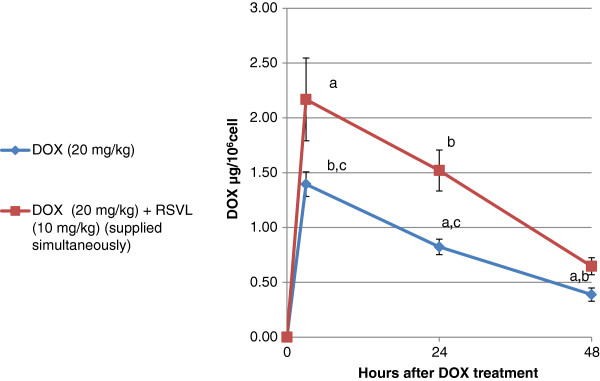
**Effect of resveratrol treatment on the doxorubicin level in Ehrlich ascites cell.** Doxorubicin was injected (20 mg/kg i.p.) in tumor-bearing mice treated with 10 mg /kg,i.p. RSVL in simultaneous manner (red box) or saline (blue diamond suit). Each data represents the mean ± S.D. of six mice. ^a^Significantly different from DOX after 3 hours of treatment at P-value ≤ 0.05. ^b^Significantly different from DOX after 24 hours of treatment at P-value ≤ 0.05. ^c^Significantly different from DOX after 48 hours of treatment at P-value ≤ 0.05.

Figure 2 shows the cellular level of DOX in Ehrlich ascites cells after a single dose of DOX (20 mg/kg,i.p.) and/or RSVL (10 mg/kg,i.p.). RSVL significantly increased the cellular level of DOX at all the time points tested, the maximum level appeared 3 hours after treatment. Forty-eight hours after treatment the levels of DOX decreased but still higher after combination therapy (1.7 fold more). DOX cellular accumulation ration were 1.55, 1.85 and 1.67 after 3, 24 and 48 hours after DOX treatment, respectively.

### Effect of DOX and/or RSVL treatment on cell cycle phase progression in Ehrlich cells

**Figure 3:**
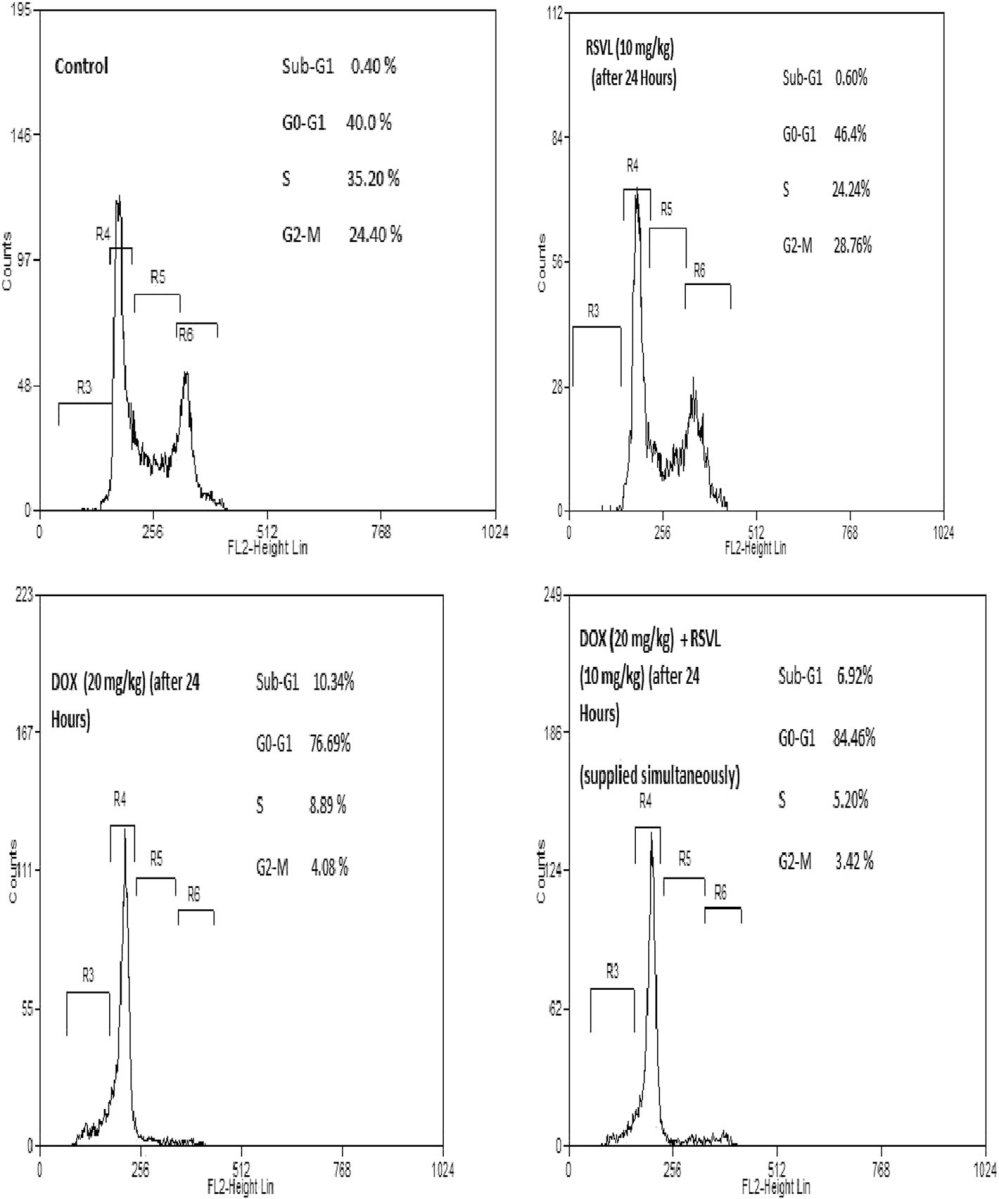
Effect of DOX (15 mg/kg) and/or RSVL (10 mg/kg) on cell cycle phase distribution of Ehrlich ascites cells.

Treatment with RSVL induced accumulation of cells in G_0_/G_1_ phase after 24 hours, while DOX treatment showed 17 fold increase in the number of cells in G_0_/G_1_ phase with less number of cells in S phase (Figure 3). Simultaneous treatment of RSVL with DOX resulted in increased number of cells in G_0_/G_1_ phase (about 85%) with reduced number of cells in S phase compared to DOX or RSVL.

**Figure 4:**
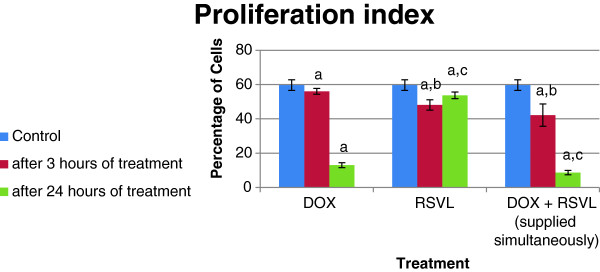
**Effect of DOX and/or RSVL on the proliferation index. **^a^Significantly different from Control at P-value ≤ 0.05. ^b^Significantly different from DOX after 3 h of treatment at P-value ≤ 0.05. ^c^Significantly different from DOX after 24 h of treatment at P-value ≤ 0.05.

Three and twenty –four hours after DOX treatment the proliferation index (S phase + G2/M phase) inhibited by about 6 and 78%, respectively, whereas concomitant treatment with RSVL reduced the proliferation index significantly by about 29 and 85%, respectively (Figure 4).

### Protective effect of RSVL against DOX-induced cardiotoxicity

**Figure 5:**
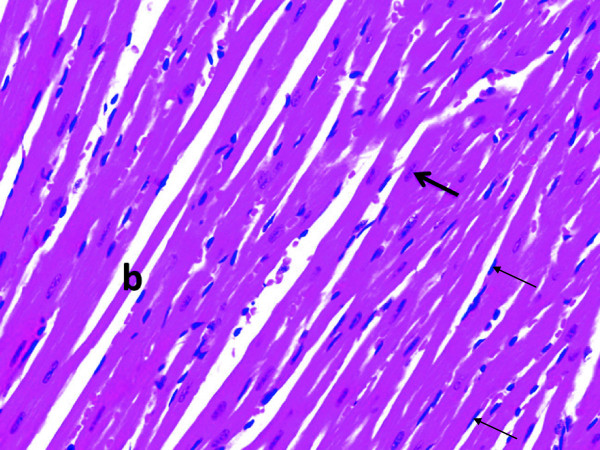
**A photomicrograph of the myocardium of a normal rat showing branching cardiac muscle fibers (b) with central oval euchromatic nuclei (thick arrow).** The fibroblast in the endomycium revealed deeply stained flat nuclei (thin arrows). H&E × 400.

**Figure 6:**
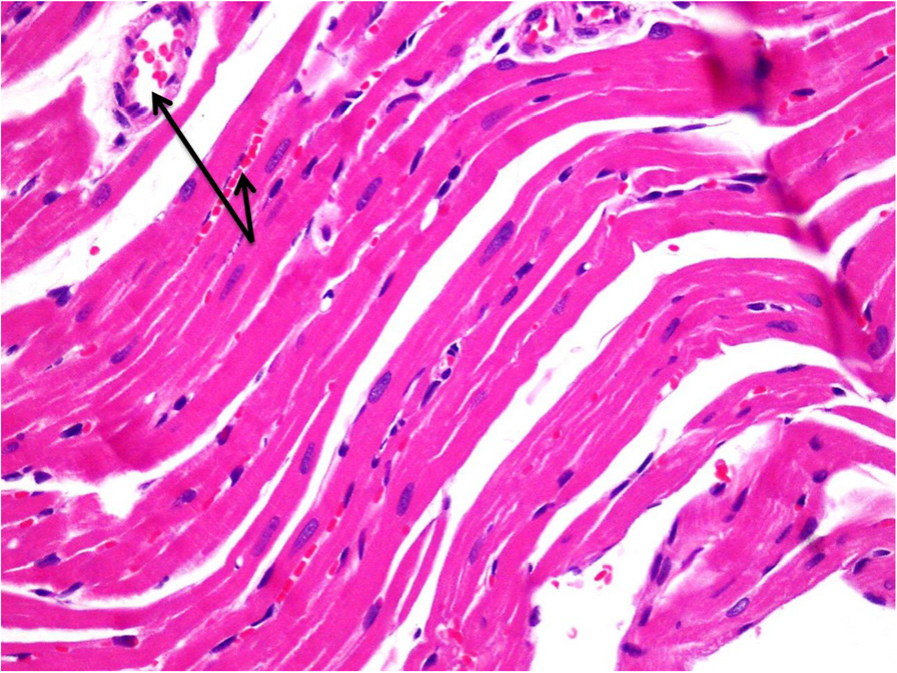
**A photomicrograph of the myocardium of a rat given RSVL (10 mg/kg,i.p.) showing a general architecture almost similar to group I (control) except for slight congestion of the blood vessels (arrows).** H&E × 400.

**Figure 7:**
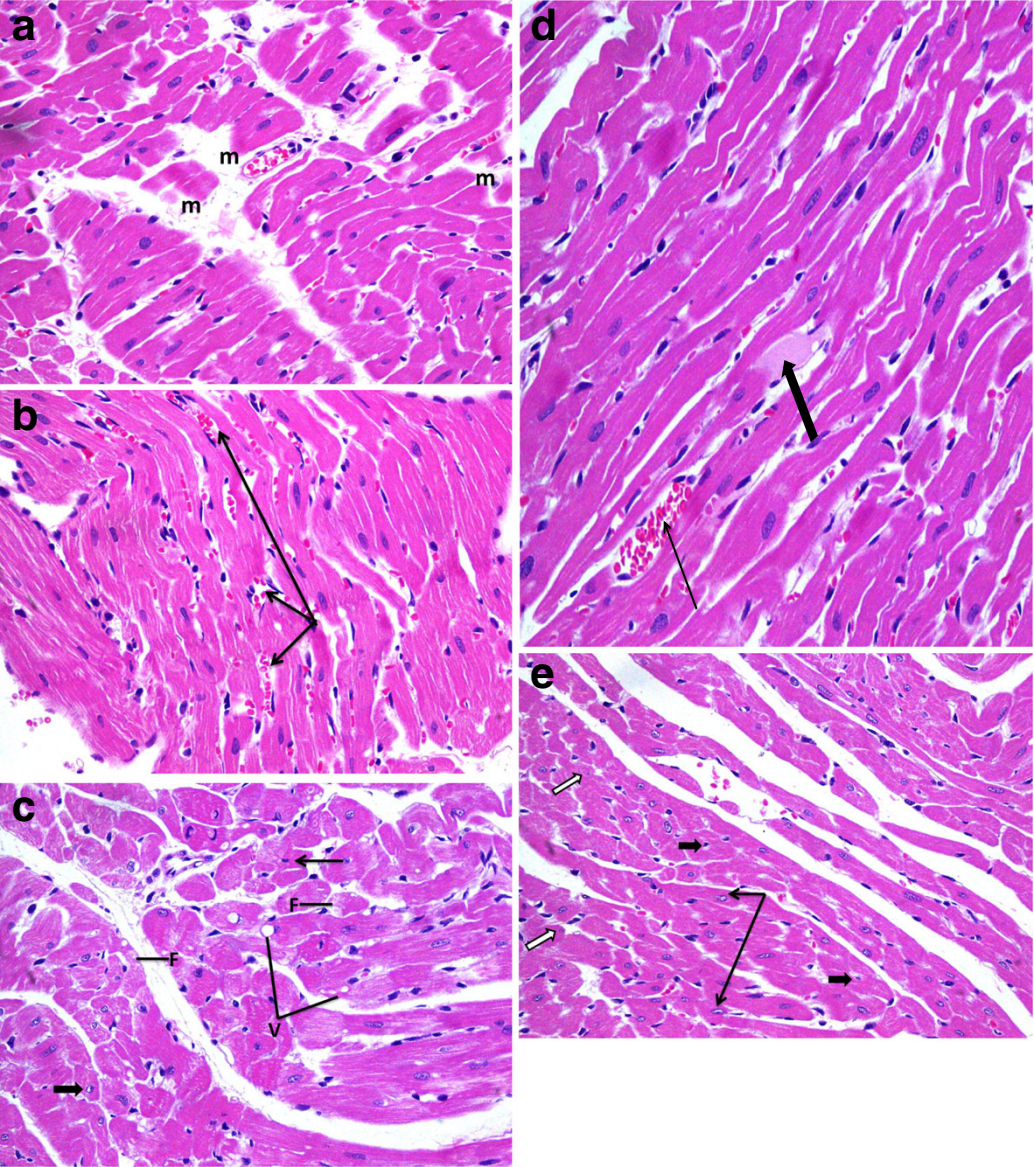
**A photomicrograph of the myocardium of a rat given DOX ( 20 mg/kg,i.p.) a :showing areas of myocytolysis(m).** H&E × 400. **b**: showing congestion of blood vessels (arrows). H&E × 400. **c**: showing myocytes with cytoplasmic vacuolization (V) or fragmentation (F). Some nuclei were pyknotic (thin arrow) while others revealed chromatin margination (thick arrow). H&E × 400. **d**: showing hyalinization of the muscle fiber (thick arrow). Notice the congested blood vessel (thin arrow). H&E × 400. **e**: showing chromatin margination of some nuclei (black arrows)while others were pyknotic (white arrows). Areas empty of muscle fibers around the pyknotic nuclei were evident (thick arrow). H&E × 400.

**Figure 8:**
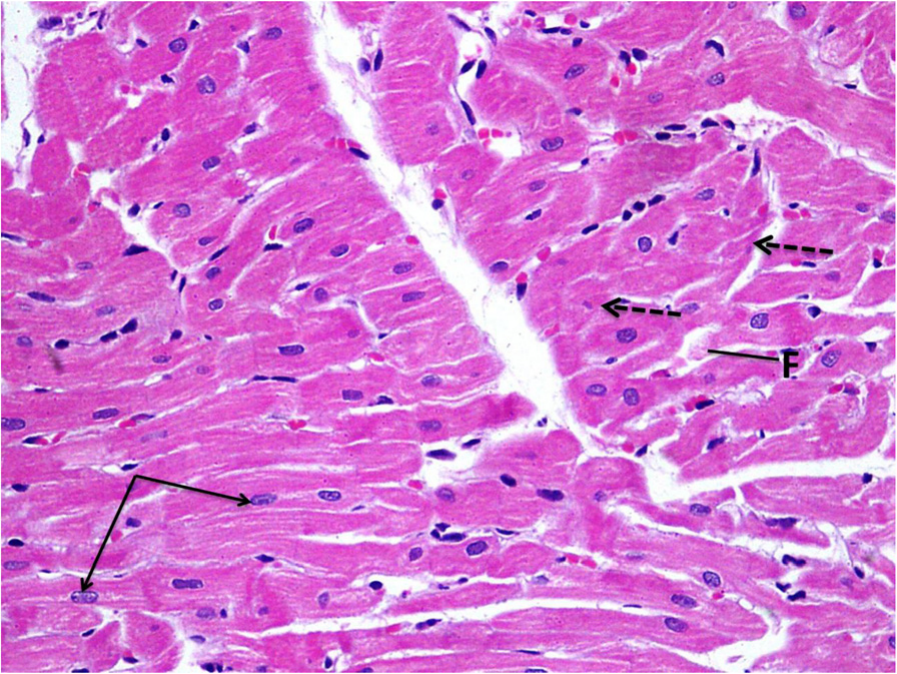
**A photomicrograph of the myocardium of a rat given RSVL (10 mg/kg,i.p.) concomitantly with DOX showing normal muscle fibers with their central oval nuclei (black arrows).** Still some pyknotic nuclei (dashed arrows) and fragmentation (F) of the muscle fiber were revealed H&E × 400.

Light microscopic examination of heart section of albino rats after a single dose of RSVL (10 mg/kg,i.p.) showed a general architecture almost similar to control (Figures 5 and 6). However, DOX (20 mg/kg,i.p.) treated animals showed area of myocytolsis (Figure 7a) with congestion of blood vessels (Figure 7b), cytoplasmic vacuolization or fragmentation (Figure 7c). In addition there was hyalinization of the muscle fiber (Figure 7d) and chromatin margination of some nuclei while others were pyknotic (Figure 7e). Concomitant administration of RSVL ( 10 mg/kg,i.p) with DOX showed normal muscle fibers with central oval nuclei and some pyknotic nuclei, while fragmentation of the muscle fiber were revealed (Figure 8).

## Discussion

Doxorubicin is an excellent antitumor drug for treating several types of solid tumor, leukemia and lynphomas. However, acute or chronic toxicity is the dose limiting factor whereas, acute cardio toxicity represented mainly by transient symptoms, such as arrhythmias, while chronic toxicity can develop into irreversible cardiomyopathy, which affects approximately 30-40% of the patients who receive 500 mg/mm^2^ total dose [1]. In the current study we investigated the modulatory effect of a natural product RSVL on the cytotoxic activity of DOX against the growth of Ehrlich ascites carcinoma in mice. At the same time, its protective effect against DOX-induced cardiotoxicity was investigated. In this study, RSVL enhanced the cytotoxic activity of DOX against the growth of Ehrlich ascites carcinoma cells. Treatment of tumor bearing mice with RSVL + DOX showed 7 –fold increase in long-term survival in comparison to DOX treated mice alone (Table 1 and Figure 1). RSVL is known to has antitumor activity in vitro [9]. Our study showed high cellular level of DOX concentrations in Ehlrich cells when RSVL was concomitantly administered with DOX (Figure 2). There was an increase in DOX accumulation ratio for cells treated with DOX and RSVL. The increase in DOX cellular uptake inside Ehrlich cells may be explained based on inhibition of P-glycoprotein that plays very important role in the absorption, distribution, and elimination of DOX and thus determine its efficacy and toxicity [13,14]. Supporting the previous work, cell cycle analysis showed that RSVL decreased the proliferation index (S phase + G_2_/M phase) of cells treated with DOX to 29 and 85% compared with 6 and 78% in cells treated with DOX alone, 3 and 24 hours after DOX treatment, respectively. This agrees with the uptake study, where DOX uptake was at highest level 3 hours after DOX treatment. Al-Shabanh et al. [15] reported similar results where the peak of DOX uptake in tumor was observed three hours after DOX treatment. Recently, Osman et al. [9], showed high DOX concentration in MCF-7 cells when concomitantly administered with RSVL. It has been shown that P388 leukemia cells synchronized in S and G_2_/M phases were more sensitive to DOX than cells in G1 phase [16], however, our study showed accumulation of Ehrlich cells in G1 phase by RSVL,DOX and their combination with significant decrease in number of cells in S phase which may be due inhibition of the enzyme used for DNA replication [17-19] or increase expression of positive G_1_/S regulators, such as cyclin D1 and cyclin E which are responsible for S phase entry [20,21]. Although our finding is somehow contradictory to other results reported a significant accumulation of some cancer cells in S-phase after RSVL treatment [22] This could be due to different cell lines used and the deregulation of expression and/or activities of different isoforms of cyclins and CDKs [23].

RSVL which is known to display antitumor activity [24], has been shown to have potent cardioprotective effect. This was observed in our study, where DOX treatment DOX showed cardiac myocytes with cytoplasmic vacuolization or fragmentation. Some nuclei were pyknotic while others revealed chromatin margination (Figure 7a, 7b, 7c). In the presence of RSVL cardiac myocytes showed normal muscle fibers with their central oval nuclei in addition to some pyknotic nuclei and fragmentation of the muscle fiber (Figure 8). In animal models of cardiovascular disease, RSVL has been shown to protect the heart from ischemi reperfusion injury, reduce blood pressure and cardiac hypertrophy on hypertensive animals and has been shown to slow progressionof athrosclerosis [25]. The exact mechanism of cardioprotective is not understood, but it could be due to inhibition of DOX-induction of the rapid increase in ROS accumulation in cardiac cell mitochondria [26] by increasing superoxide dismutase activity [27], suggesting that the antioxidant properties of resveratrol may play a role in its cardioprotective effects. However, antioxidant therapies have failed to produce satisfactory results in clinical trials [28], casting doubt on the notion that the inhibition of oxidative stress is the only mechanism responsible for the cardioprotective effects of resveratrol which may need further investigations. In conclusion, this study suggests that RSVL treatment increased the cytotoxic activity of DOX against the growth of Mammary tumor in vivo and could be as a promising cardioprotective agent against DOX-induced cardiotoxicity.

## Competing interests

The authors declare that they have no competing interest.

## Authors’ contributions

AMO, SEA, OMA, HMA, ZAD, MNA and OHO sharing in experimental work and writing the manuscript. MFA did the flow cytometric analysis and interpreted the results and WSR investigated the pathological changes in the hearts together with NMA. All authors read approved the final manuscript.
